# Hierarchical NiO nanobelt film array as an anode for lithium-ion batteries with enhanced electrochemical performance

**DOI:** 10.1039/c8ra03599g

**Published:** 2018-07-25

**Authors:** Ning Hu, Zheng Tang, Pei Kang Shen

**Affiliations:** Collaborative Innovation Center of Sustainable Energy Materials, Guangxi University China pkshen@gxu.edu.cn; Guangxi Key Laboratory of Electrochemical Energy Materials, Guangxi University China; State Key Laboratory of Processing for Non-ferrous Metal and Featured Materials, Guangxi University Nanning 530004 P. R. China

## Abstract

In this study, an ultrathin 2-dimensional hierarchical nickel oxide nanobelt film array was successfully assembled and grown on a Ni substrate as a binder-free electrode material for lithium ion batteries. In the typical synthesis process, the evolution of the nickel oxide array structure was controlled by adjusting the amount of surfactant, duration of reaction time and hydrothermal temperature. By virtue of the beneficial structural characteristics of the nanobelt film array, the as-obtained NiO array electrode exhibits excellent lithium storage capacity (1035 mA h g^−1^ at 0.2C after 70 cycles and 839 mA h g^−1^ at 0.5C after 70 cycles) for LIBs. This excellent electrochemical performance is attributed to the nanobelt film (3–5 nm thickness) array structures, which have immense open spaces that offer more Li^+^ storage active sites and adequate buffering space to reduce internal mechanical stress and shorten the Li^+^ diffusion distance. Additionally, this array structure is designed to achieve a binder-free and non-conductive additive electrode without complex coating and compressing during the electrode preparation process.

## Abstract 2

In this study, an ultrathin 2-dimensional hierarchical nickel oxide nanobelt film array was successfully assembled and grown on a Ni substrate as a binder-free electrode material for lithium ion batteries.

## Graphical abstract

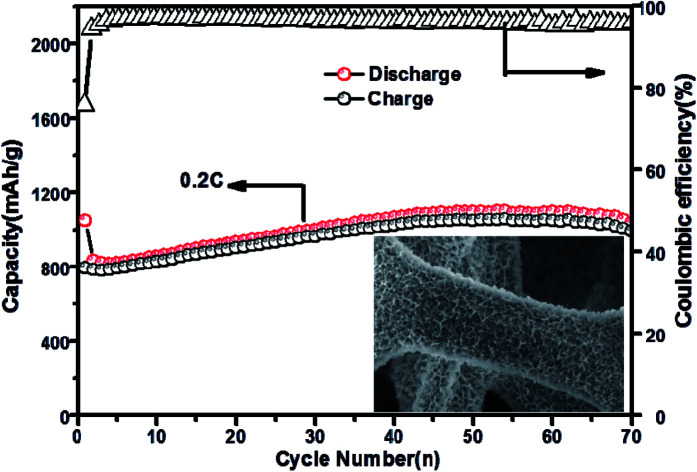

## Introduction

The issues of shortage of global fossil fuel sources and deteriorating environment have attracted worldwide attention over the past decades. Hence, there is an urgent desire to seek renewable energy sources that can replace traditional fossil fuel and improve the quality of the global environment. Nowadays, rechargeable batteries are conducive to relieving the energy and environment issues. For example, lithium-ion batteries (LIBs), as highly efficient clean energy storage devices, have wide applications in portable electronic devices such as cell phones, laptops, digital cameras and electronic vehicles as well as in energy storage systems used in our daily lives. This is due to their many excellent distinctions, including high energy density, long cycle life, environmental friendliness, absence of memory effect, high output voltage, and minor self-discharge.^1–6^ It is well known that commercial graphite is currently applied as representative anode materials for lithium ion batteries, but graphite has a lower lithium storage capacity and only 372 mA h g^−1^ theoretical capacity and hence, fails to meet the high-energy and power-density demands of electric vehicles and large-scale energy storage grid fields.^7^ Therefore, in order to satisfy practical needs, we must develop both high-energy and long-cycle electrode materials. Recently, transition metal oxides (TMOs) have attracted considerable attention in the energy storage field and are promising next generation electrode materials for lithium ion batteries because most TMOs have high theoretical specific capacity (above 600 mA h g^−1^), which is 2–4 times higher than that of the carbon/graphite materials.^8,9^ TMOs have been extensively investigated to develop high lithium storage electrodes for replacing graphite-based materials. Although traditional powder transition metal oxide materials used as anode electrode materials for lithium ion batteries have higher capacity, their cycling stability and rate performance do not satisfy the application requirements. Most transition metal oxides are semiconductors or even insulators, so they exhibit poor electrical conductivity and experience large volume changes, which cause the active materials to deteriorate during the deep lithium insertion and delithiation, resulting in poor cycling stability and rate performance of lithium ion batteries.^10,11^ Because of these drawbacks, the development of TMOs for application in lithium ion batteries is greatly restricted. Therefore, in the past decades, many researchers have been devoted to study TMO ordered nanostructure array electrode materials directly grown on metal substrates to bypass the abovementioned issues^12,13^ for improving the electrochemical performance of LIBs. Such materials include nanowalls,^14^ nanosheets,^15^ nanobelts,^16^ nanotubes^17^ and nanowires.^18^ Metal oxide based binder-free array electrodes can be developed, offering higher output voltage and fast energy storage.^19–23^

In this article, we concentrate on the binder-free array electrode materials for improving the electrochemical performance of LIBs. By a simple hydrothermal method, nickel oxide (NiO) nanobelt film array was uniformly synthesized on nickel foam substrate, which works as both current collector and structure support. Among the TMOs, NiO has been strongly explored owing to its high theoretical specific capacity (∼718 mA h g^−1^), low material cost and nontoxicity.^24^ The as-obtained NiO nanobelt film array was synthesized for the first time under the influence of SDS. The unique nanobelt array can prevent aggregation or leaching of the active materials. Additionally, the novel nanobelt film array structure has huge open spaces, contributing to the acceleration of electron transport and shortening of the pathway of ion transport. Moreover, the assembled array electrode consists of binder-free and nonconductive additives, which can reduce cost and avoid the complicated electrode production process. Based on these results, it is believed that the nickel oxide nanobelt array is a promising next generation lithium ion battery anode material.

## Experimental section

### Preparation of NiO nanobelt array structure

The NiO nanobelt film array was synthesized *via* a novel self-sustained cycle of hydrolysis and etching (SCHE) route as previously reported.^25^ In the typical synthesis process of the NiO nanobelt film array, Ni foam (*d* = 14 mm) substrates were ultrasonicated in 3 M HCl, ethanol and DI water for 15 min to remove surface impurities before the synthesis. In addition, 0.025 M Ni(NO3)_2_·6H_2_O and 0.01 g sodium dodecyl sulfate (SDS) were dissolved in 20 mL DI water under vigorous stirring. Then, the uniform green solution was transferred to a 50 mL Teflon lined stainless-steel autoclave. Subsequently, the cleaned nickel foam substrate was immersed horizontally at the bottom of the autoclave. The autoclave was sealed and maintained in an oven at 180 °C for 12 h. After the autoclave reaction was completed, the autoclave was cooled to room temperature. The nickel foam was taken out from the reaction solution, washed with ethanol and DI water to remove the impurities, and finally dried at 80 °C in an oven.

The NiO nanobelt film arrays were prepared by post-annealing of the Ni(OH)_2_ precursor. The as-obtained Ni(OH)_2_ grown on nickel foam substrate was heated to 400 °C for 2 h in a tube furnace at a low heating rate of 2 °C min^−1^ under nitrogen atmosphere.

### Physical characterization

The crystallographic information and phase structure of the as-prepared samples was tested by a D/Max-III X-ray diffractometer (Rigaku Co., Japan) with Cu Kα radiation, a voltage of 30 kV, and a current of 30 mA. The scan range was 2*θ* = 10° to 90° and the scan rate was 10° 2*θ* min^−1^. A field-emission scanning electron microscope (FESEM; SU8820, Hitachi Co., Japan) and transmission electron microscope (TEM; Titan ETEM G2 80-300, FEI Co., USA) were employed to examine the morphology and conduct elemental analysis of the products.

### Electrode preparation and measurements

The NiO nanobelt film arrays directly grown on Ni substrates were used as the working electrode without being any compressed. The mass of the as-prepared NiO nanobelt film arrays were measured using a microbalance by weighing the product before and after ultrasonication of the as-prepared NiO nickel foam substrate in 3 M HCl solution. The measured mass of the NiO nanobelt film arrays on the nickel foam substrate was about 1.3–1.5 mg cm^−2^. Prior to battery assembly, the NiO nanobelt film array electrodes were dried in a vacuum oven at 80 °C for 12 h. In the half-cell configuration, metallic lithium foil was used as the counter electrode. In addition, 1 M LiPF_6_ in ethylene carbonate (EC)/dimethyl carbonate (DMC)/diethyl carbonate (DEC) (1 : 1 : 1 v/v/v) was used as the electrolyte and polypropylene (PP) microporous membrane (Cellgard 2400) was used as the separator. The coin cells (CR2032 type) were assembled in an argon-filled glove box, in which H_2_O and O_2_ ingredients were maintained below 0.1 PPM. For comparing the electrochemical performance of powder and nanobelt film array electrode, we prepared the powder electrode comprising 80% commercial NiO powder nanoparticle, 10% conductive additive (super p) and 10% binder (PVDF). The assembled cells were set aside for 24 hours before the electrochemical measurements. The cyclic performance and rate measurements were tested on a battery-testing system (Shen Zhen Neware Battery Co; China) with galvanostatic charge–discharge between 0.01 V–3 V (*vs.* Li/Li^+^). The cyclic voltammograms (CVs) were performed at a scan rate of 0.1 mV s^−1^ from 0.01 V to 3 V, and the electrochemical impedance spectroscopy (EIS) measurements were carried out on an IM6 electrochemical workstation (Zahner-Elektrik, Germany) with an amplitude of 5 mV from the frequency range of 100 kHz to 10 mHz.

## Results and discussion

### Material characterization

**Scheme 1:**
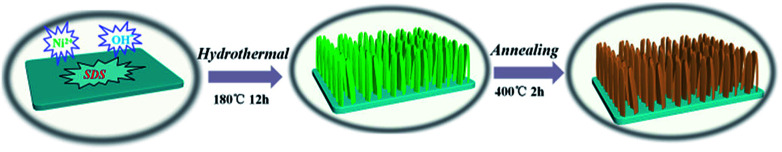
Schematic illustration of the formation processes of the hierarchical NiO nanobelt film arrays.

**Fig. 1:**
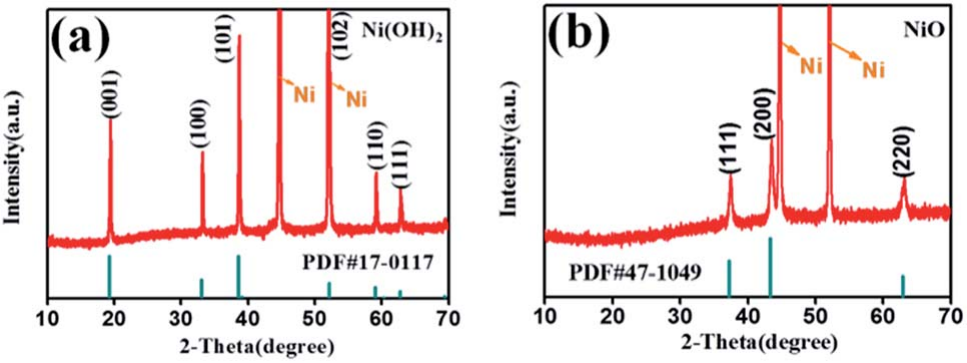
XRD pattern of the (a) Ni(OH)_2_ and (b) NiO nanobelt film array grown on Ni substrate.

Scheme 1 illustrates the fabrication process of NiO nanobelt film array. The Ni(OH)_2_-precursor was synthesised by a hydrothermal method at 180 °C for 12 h, and the NiO nanobelt film arrays were obtained by post-annealing the Ni(OH)_2_-precursor at 400 °C for 2 h under a nitrogen atmosphere. The XRD patterns of the Ni(OH)_2_-precursor and the corresponding NiO nanobelt film array materials on nickel foam substrate are shown in Fig. 1a and b, respectively. All of the diffraction peaks of the Ni(OH)_2_-precursor and corresponding NiO are in good agreement with standard crystallographic data and can be unambiguously indexed and confirmed. Excluding three strong peaks from the Ni conductive substrate, the as-prepared Ni(OH)_2_ sample shows diffraction peaks at 19.3°, 33.1°, 38.5°, 52.1°, 59.1°, and 62.7°, which correspond to the (001), (100), (101), (102), (110) and (111) lattice planes, indicating the Ni(OH)_2_ structure (PDF#17-0117). The annealed NiO nanobelt film array displays three distinct peaks at 37.3°, 43.3° and 62.9°, corresponding to the crystal planes of (111), (200) and (220) of the cubic NiO (PDF#47-1049). The strong and narrow peaks manifested the good crystallinity of the as-prepared NiO. In addition, the nickel nitrate precursor is completely converted to crystalline nickel oxide because no other miscellaneous peak is observed in the pattern.

**Fig. 2:**
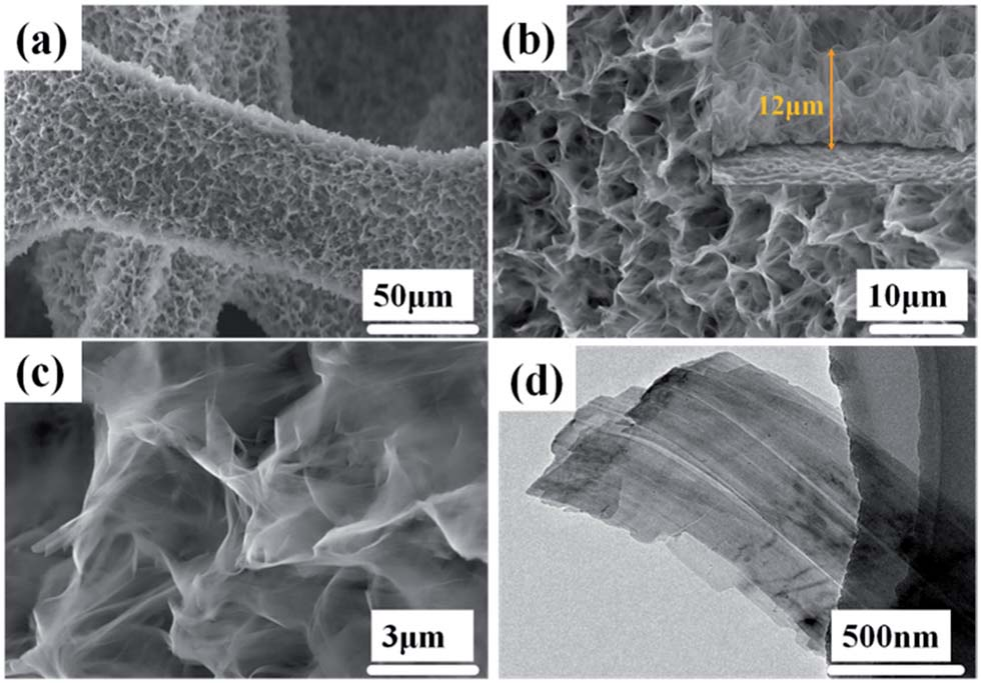
Morphological and structure characterization of hierarchical Ni(OH)2 nanobelt film array: (a)–(c) SEM images, (d) TEM image.

The morphologies and structures of the Ni(OH)_2_-precursor and NiO nanobelt film arrays were observed by SEM and TEM techniques. The images were investigated to explore the morphologies of the Ni(OH)_2_ nanobelt film array structure after hydrothermal reaction in the 0.025 M nickel nitrate solution and 0.01 g SDS for 12 h at 180 °C. As shown in Fig. 2, large-scale uniform porous nanobelt film arrays can be clearly seen on both sides of the nickel foam substrate (Fig. 2a). Moreover, the well-organized and interconnected nanobelt film frame adhered firmly on the Ni substrate, as clearly observed in Fig. 2b and c. The average height of the nanobelt array was about 10–15 μm from the small side view (inset of Fig. 2b), and the Ni(OH)_2_ array structure consists of many interlacing sheets, with an average width of over 500 nm, based on the TEM image (Fig. 2d).

**Fig. 3:**
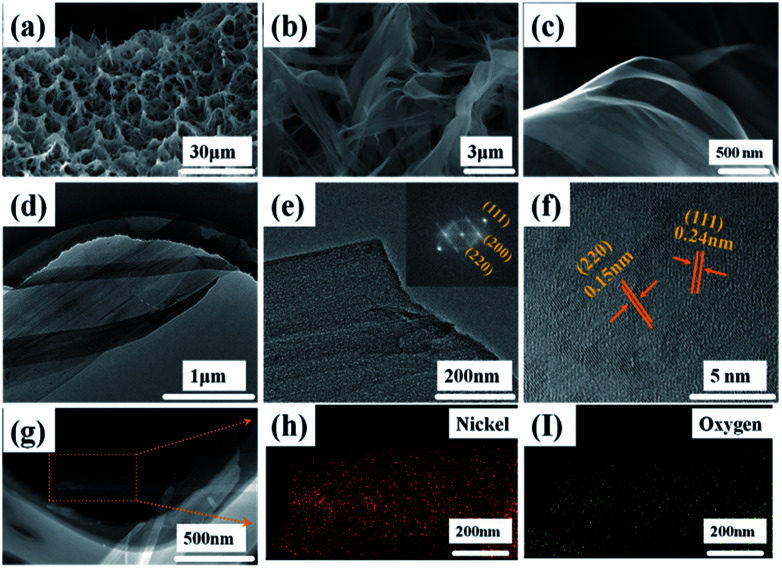
Morphological and structural characterization of hierarchical NiO nanobelt film array structure: (a–c) SEM images, (d) TEM, (e) HR-TEM and FTT pattern, (f) HR-TEM (g–i) EDX pattern of NiO nanobelt film.

The NiO array structure can be investigated after thermal dehydration of the Ni(OH)_2_ precursors by SEM and TEM (Fig. 3). It can be clearly observed that the NiO array structure is more shriveled, as shown Fig. 3a which is attributed to the nickel hydroxides loss of water molecules after the post-heating process; the average porosity size is about 6.67 μm. Fig. 3b displays the intricate and connected NiO nanobelt film interwound with each other, and this open framework provides a huge space for sufficient contact of the electrolyte and active materials, which helps reduce the damage from internal stress to maintain the structural stability. The transparent and extremely thin film structure can be clearly seen in Fig. 3c. The TEM images were used to further investigate the morphology and crystal structure of the as-prepared NiO nanobelt film arrays. Fig. 3d and e show that the TEM images consist of a few transparent films stacked together, clearly corroborating the SEM images. Moreover, the lattice spacing was confirmed to be 0.24 nm and 0.15 nm on the basis of the high-resolution TEM images, as shown in Fig. 3f, which is consistent with the (111) and (220) interplanar spacing of NiO. The corresponding FFT image (inset of Fig. 3e) displays single crystals of NiO and the diffraction spots are clearly attributed to (111), (200) and (220) crystal facets, which are consistent with the XRD result. The EDX spectra (Fig. 3g–i) demonstrate the mapping analysis of nickel and oxygen elements, which are uniformly distributed in the nanobelt film array structure.

### Morphological evolution of Ni(OH)_2_ nanobelt arrays

In order to study the evolution of Ni(OH)_2_ morphology and structure, the influencing factors, namely, surfactant, time and temperature were investigated.

**Fig. 4:**
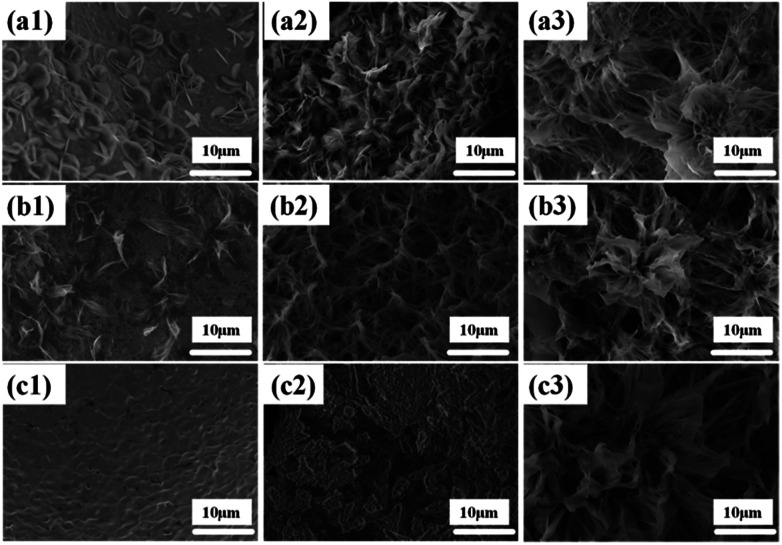
Morphological and structural characterization of Ni(OH)_2_ at different SDS: (a1) 0 g (a2) 0.005 g (a3) 0.02 g; at different reaction times: (b1) 6 h (b2) 18 h (b3) 24 h; at different reaction temperatures: (c1) 140 °C (c2) 160 °C (c3) 200 °C.

The effect of sodium dodecyl sulfate (SDS) as a surfactant was investigated to adjust the morphology of the Ni(OH)_2_ arrays. The SEM images (Fig. 4a1–a3) show the synthesis process of the Ni(OH)_2_ arrays with different concentrations of sodium dodecyl sulfate. When SDS is not added to the aqueous solution, it was observed that a few nanosheets unhomogeneously grew on the Ni foam substrate, as shown in Fig. 4a1, and the width of the as-obtained Ni(OH)_2_ nanosheets was about 5 μm. The reason that only a few Ni(OH)_2_ nanosheets were grown on the Ni substrate is that the energy to sustain grain growth was insufficient. Subsequently, as the amount of the added SDS surfactant was increased to 0.005 g and 0.02 g, the Ni(OH)_2_ nanosheets slowly grew and transformed to a mass of nanobelt arrays, as shown in Fig. 4a2 and a3, respectively.

The hydrothermal reaction time plays a vital role in the process of forming a uniform nanobelt film array on the substrates. The SEM images of Ni(OH)_2_ nanobelt film structure at different reaction time intervals can contribute to understanding the formation mechanism of Ni(OH)_2_ nanobelt film arrays. As shown in Fig. 4b1, the materials, prepared with the reaction time of 6 h, were assembled and formed the preliminary structure, which consists of a single piece of film. With the increase in the reaction time, the porous nanobelt arrays gradually overgrow and cover the Ni substrate at 18 h, as shown in Fig. 4b2. Moreover, the Ni(OH)_2_ nanobelt film arrays grew wider and longer. It can be clearly seen that petal-like arrays are formed as the reaction time was prolonged to 24 h (Fig. 4b3). The structure has an average height of about 20 μm. These observations indicate that extension of reaction time conduces the growth of crystal particles and controls the morphologies of the resultant film arrays.

In the synthesis of Ni(OH)_2_ nanobelt film array structures, temperature is also a very important factor. Fig. 4c1 demonstrates that no material growth on the Ni substrate occurs at 140 °C, because crystal particle growth is not achieved at this temperature. When the temperature was increased to 160 °C, drastic agglomeration of the Ni(OH)_2_ crystal particles can be clearly seen on the Ni substrate surface, as shown in Fig. 4c2, which illustrates crystal particle growth above 160 °C. With the reaction temperature increasing to 200 °C, Fig. 4c3 displays that Ni(OH)_2_ crystals can obtain sufficient energy to rapidly grow and turn into nanobelts under the action of the surfactants.

In summary, to control the thickness, we can adjust the reaction time, temperature and SDS concentration. With the increase in reaction time, temperature and SDS concentration, the thickness of the film will also increase.

### Electrochemical performance of NiO nanobelt array for LIBs

**Fig. 5:**
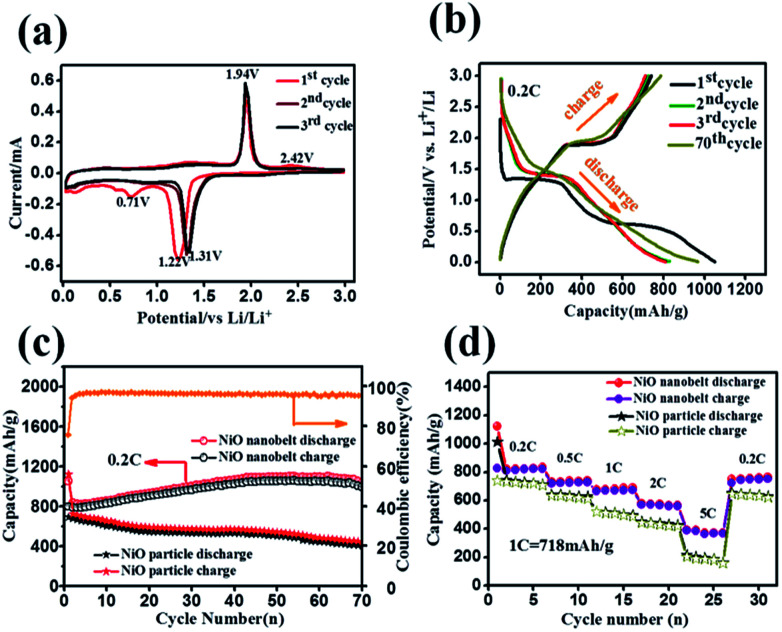
Electrochemical performance of the NiO nanobelt array as a LIB anode on foam nickel substrate: (a) cyclic voltammograms curves at a scan of 0.1 mV s^−1^, (b) the galvanostatic discharge–charge voltage profiles at 0.2C, (c) cycling performance at 0.2C, (d) rate capabilities at various rates (1C = 718 mAg^−1^).

Hierarchical nanostructure NiO nanobelt film arrays grown on Ni substrate were applied to the lithium ion battery, which showed excellent electrochemical performance.^26^ NiO nanobelt array shows excellent promise to become the next generation anode material of lithium ion batteries. To illustrate the excellent electrochemical properties of the NiO nanobelt film arrays, a series of electrochemical tests were conducted by cyclic voltammetry measurements and galvanostatic discharge–charge tests. Fig. 5a shows the charge–discharge profiles of the NiO nanobelt film array electrode of the first three cycles at a scan rate of 0.1 mV s^−1^ from 0.01 V to 3 V (*vs.* Li/Li^+^) range. The strong reduction peaks shown in the first cycle were different from those in the second and third profiles, which is mainly due to the decomposition of the electrolyte to form the solid electrolyte interphase (SEI) layer and Li_2_O.^27^ The two shoulder reduction peaks located at ∼1.22 V and ∼0.71 V were detected in the first discharge curve, and the underlying reduction reaction was most likely the decomposition of NiO to Ni^0^, and the simultaneous formation of Li_2_O.^28^ For the first charge process, the oxidation peak was observed at ∼1.94 V, corresponding to oxidation of NiO to Ni^0^ and Li_2_O decomposition.^29^ The reduction peak shifted to ∼1.31 V owing to structural rearrangement, resulting in irreversible capacity loss in the second and third process.^30^ In the subsequent scans, the cyclic voltammetry curves showed a high degree of coincidence, which indicates a high degree of cyclability and stability of the battery. The reversible reaction can be summarized as follows:^31^NiO + 2Li^+^+ 2e^−1^ ↔ Ni + Li_2_O

**Fig. 6:**
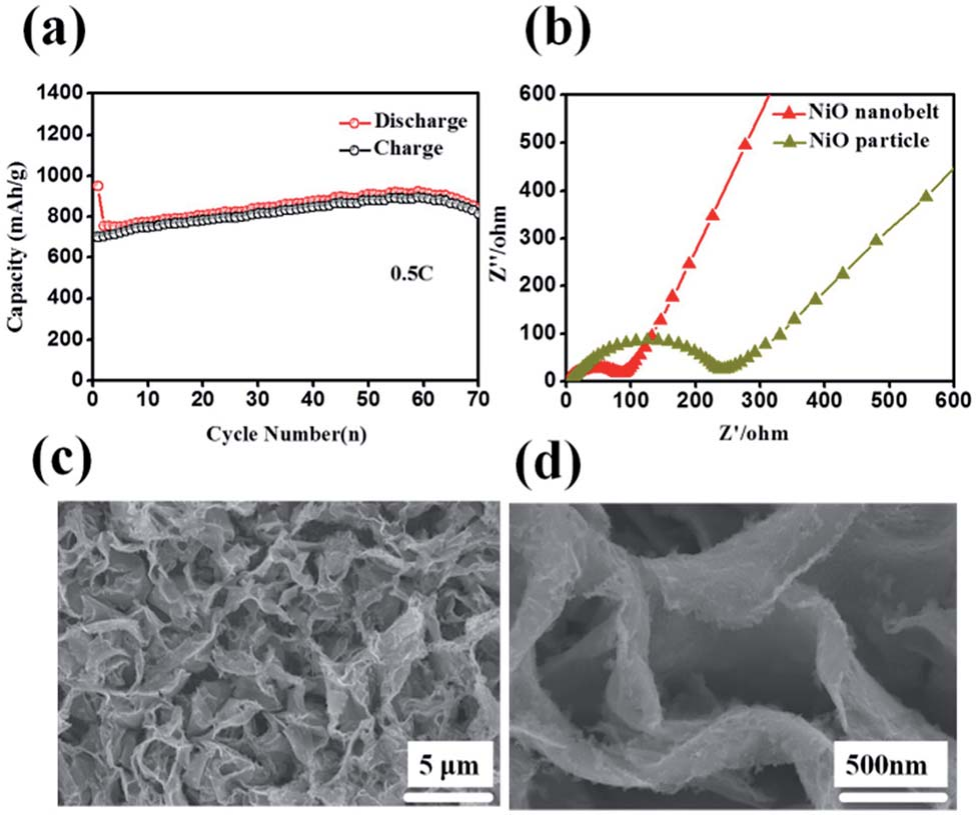
(a) Cycling performance at 0.5C (b) Nyquist plots of the NiO nanobelt array electrode (c) and (d) SEM images of the NiO nanobelt array after 70 cycles at 0.2C.

Representative galvanostaic discharge–charge was tested in the voltage range of 0.01 V–3.0 V at a current density of 0.2C (1C = 718 mA h g^−1^), as shown in Fig. 5b. The first discharge cycle voltage platform at ∼1.22 V and ∼0.7 V was consistent with CV measurements. However, the two discharge potential plateaus move up ∼1.31 V from the 2^nd^ cycle with good superposition, which illustrates that a steady SEI layer could be formed in the first cycle.^32^ The initial discharge and charge capacity of the NiO nanobelt film array electrode reached up to 1050.7 mA h g^−1^ and 795.3 mA h g^−1^, respectively. Moreover, this value exceeds the NiO theoretical capacity (∼718 mA h g^−1^). Compared to the NiO nanoparticles electrode, the NiO nanobelt film array electrode as LIB anode shows superior cycling performance and rate performance. In order to further investigate the properties of the NiO nanobelt film electrode, its cycling performance over 70 cycles of discharge and charge at 0.2C rate was determined (Fig. 5c). It can be observed that the NiO nanobelt electrode shows higher capacity and coulombic efficiency. The corresponding initial coulombic efficiency reached up to 75.69%, which is higher than the nickel oxide powder. It can be seen that the capacity of the NiO array electrode gradually increases and then remains at 1034.7 mA h g^−1^ after 70 cycles, and the specific capacity only decreased by about 1.46% (relative to initial discharge capacity). This performance of the NiO nanobelt film array is superior to that obtained in previous reports, as shown in Table 1.^26,33–40^ The specific capacity continuously increased due to the constant activation of active material and the reversible generation of a polymeric gel-like layer originating from kinetic activation in the electrode,^41,42^ which is characteristic for anode materials. For the NiO powder nanoparticles, the discharge and charge capacity quickly decreased to only 440.1 mA h g^−1^ and 401.3 mA h g^−1^, respectively, after 70 cycles at the corresponding 0.2C rate, which was due to the particle structure suffering severe damage, suggesting that the NiO nanobelt film array structure is more beneficial for ion transport and contact between electrode and electrolyte interface because of its superior geometric characteristics.^43–45^ Most importantly, the as-fabricated NiO nanobelt film array approximately retained 646.1 mA h g^−1^ at 0.5C rate after 70 cycles, as shown in Fig. 6a, which indicates that the nanobelt structure still has a long cycle performance at higher C-rates.

**Table 1:** The lithium storage performance of NiO compared to composite electrodes

Materials	Method	Current density	Reversible capacity	Cycle number	Ref.
NiO nanosheets	Hydrothermal	0.2C	1043 mA h g^−1^	80 cycles	26
NiO nano octahedron	Hydrothermal	0.2C	793 mA h g^−1^	200 cycles	33
Co-doped NiO nanoflake	Hydrothermal	100 mA g^−1^	600 mA h g^−1^	50 cycles	34
NiO microspheres	Hydrothermal	500 mA g^−1^	800.2 mA h g^−1^	100 cycles	35
NiO@C	Hydrothermal	1000 mA g^−1^	739 mA h g^−1^	100 cycles	36
NiO nanorod array	Anodization	1000 mA g^−1^	705.5 mA h g^−1^	70 cycles	37
NiO nanofiber	Electrospinning	80 mA g^−1^	784 mA h g^−1^	100 cycles	38
NiO-GNS	Hydrothermal	71.8 mA g^−1^	1031 mA h g^−1^	40 cycles	39
Ni/NiO	Gas–solid oxidation	156 mA g^−1^	646 mA h g^−1^	65 cycles	40
**NiO nanobelt film array**	**Hydrothermal**	**0.2C**	**1035 mA h g** ^**−1**^	**70 cycles**	**Our work**

C-rate performance was used to evaluate electrode material properties in lithium ion batteries. Therefore, the rate performance of the NiO nanobelt film array and NiO powder particles were measured and compared at different current densities, and the results are shown in Fig. 5d. At C rates of 0.2, 0.5, 1, 2 and 5C, the NiO nanobelt film array displayed discharge capacities of 835.4, 737.6, 689.8, 565.7 and 372.8 mA h g^−1^, respectively. The corresponding capacities of the NiO powder particles were 710.8, 625.9, 494.4, 425.1 and 161.5 mA h g^−1^, respectively. Clearly, the nanobelt film array exhibits superior rate performance compared to that of the powder particles for the lithium ion battery. Moreover, the discharge capacity of 765 mA h g^−1^ can be achieved again on reversing the C rate from 5C to 0.2C, indicating that the NiO nanobelt film array structure was well preserved even after cycling at high current density. Above all, the NiO nanobelt film array electrode possesses superior electrochemical properties than NiO powder for the lithium ion battery.

Such outstanding rate performance is attributed to the NiO nanobelt film arrays grown directly on the nickel foam substrate surface forming a complete structure, which enhances contact area between the active material and the current collector and decreases the contact resistance. NiO nanobelt film arrays have more open spaces, which enable more active materials to participate in redox reaction in the electrolyte. Also, the open space array was more effective in buffering the structural change and prevented the active material aggregation in the Li^+^ insertion and extraction processes as compared with the NiO powder active materials coated and post-pressuring on copper foil. Accordingly, ion diffusion and electron transfer occur more easily in this structure, which can expedite electrochemical reaction kinetics during the discharge and charge cycles. Due to the increased contact area between the array structure and the electrolyte, the lithium ion diffusion path is reduced. As a result, excellent cycling performance and rate performance can be obtained. The Nyquist plots of the fresh NiO nanobelt film array electrode and powder NiO particle electrode are shown in Fig. 6b. The NiO nanobelt array electrode demonstrates lower charge-transfer resistance (∼70 Ω) than NiO powder particles electrode (∼270 Ω). The unique nanobelt array structure can provide more contact area between the active material and electrolyte, which results in faster electron transfer and Li^+^ diffusion during the charge and discharge processes. To determine the relationship between the excellent electrochemical performance and unique array structure, the morphological evolution of the NiO nanobelt array structure electrode was investigated after 70 cycles of discharge and charge at 0.2C, as shown in Fig. 6c and d. The integrated array structure was still well-preserved. The array structure can effectively relieve the stress of the volume change. Thus, the electrochemical performance of the electrode can be improved.

## Conclusions

In summary, we described a facile hydrothermal synthesis method for fabricating hierarchical NiO nanobelt film arrays grown on Ni substrate for high-performance lithium ion batteries. The novel hierarchical nanobelt film array structure was synthesized by adjusting and controlling the conditions of temperature and duration of the hydrothermal reaction and surfactant concentration. The unique nanobelt film array structure directly grown on Ni substrate can provide more Li^+^ storage sites and shorten electronic transmission and ion diffusion distance in the discharge and charge process, which results in improved conductivity and electrochemical properties. Moreover, the formation of the hierarchical nanobelt film structures improves the specific capacity and rate performance compared to commercial NiO powder particles. In addition, the discharge capacity of 1050.7 mA h g^−1^ can still be achieved at 0.2C after 70 cycles, and the capacity is higher than that of most transition metal oxide materials for lithium ion batteries. Due to its excellent electrochemical performance, the NiO nanobelt film array electrode can be a promising next generation anode material for lithium ion batteries.

## Conflicts of interest

There are no conflicts to declare.

## Supplementary Material
